# 

**Published:** 1894-01-13

**Authors:** 


**Notice to Advertisers and Subscribers.**

All Advertisements, Orders for Copies of The Hospital, and Business Communications should be addressed to The Publisher, *not to the Editor*, The Hospital, 428, Strand, W.C.

The Cost of Subscription, post free, is as follows:—Per week, 2½d.; per quarter, 2s. 6d.; per half-year, 5s.; per annum, 8s. 6d. Foreign:—Per Annum, 10s. 8d.; payable, in all cases, in advance.

**OUR NEW OFFICES.**

428, Strand, W.C., will henceforth be the Office of this Journal.

**Notices of Births, Deaths, and Marriages** are inserted in this column at a charge of 2s. 6d. for an announcement not exceeding four lines.

BIRTH.

Bays.—On January 4th, at 77, Finchley Road, N.W., the wife of James T. Bays, M.D. Lond., of a daughter. Australian and Indian papers, please copy.

MARRIAGE.

Langran—Powne.—On January 4th, 1894, at St. Mary's Church, Chard, Somerset, by the Rev. Prebendary Robinson, vicar, assisted by the Rev. W. S. Watson, and the Rev. T. Wood Robinson, brother-in-law of the bride, Bartholomew Langran, L.R.C.P.Ed., of Liverpool, second son of George Langran, Carlow, Ireland, to Agnes, third daughter of William Powne, L.R.C.P.Lond., and niece of Dr. P. L. Powne, Chard.

DEATH.

Good.—On January 5th, at St. Neots, Huntingdonshire, Frederick Thomas Good, M.R.C.S., &c., second son of Alfred Good, of Orpington and Moorgate Street, in his 39th year. Funeral at St. Neots, on Tuesday, at half-past Two.

**NOTICE TO CORRESPONDENTS.**

All MS., Letters, Books for Review, and other matters intended for the Editor should be addressed The Editor, The Lodge, Porchester Square, London, W.

The Editor cannot undertake to return rejected MS., even when accompanied by stamped directed envelope.

**NOTICE.—The Index for Vol. XIV. (ending Sept. 30th, 1893) is on sale at the Office of this Journal, price 2½d., post free.**

### Scraps and Gleanings.

The Government of Cape Town have appointed a Commission to inquire into the leprosy question.

Further accommodation is required for the Middlesborough Fever Hospital. A special meeting to consider the question has been called.

At the instigation and request of Lady Penrhyn, a movement has been inaugurated in Carnarvon to establish a trained nurse for the poor of that town.

The accommodation for infectious diseases cases in the area of the Metropolitan Asylums Board was increased during the last year from 1,662 beds to 4,095.

The anniversary meeting of the Salop Infirmary was recently held. The report of the directors shows that the net receipts for the year amount to £5,372.

The annual patients' tea and entertainment were held at the Sussex County Hospital in Christmas week, when the institution was gaily decorated for the occasion.

The question of the isolation hospital at Enfield is somewhat to the fore again. The site selected by the local board has not met with the approval of the ratepayers.

Tuft's College Medical School, the new venture in Boston, which is designed for male and female students, was attended by over seventy pupils during the past year.

An appeal is being made by the inhabitants of Okehampton (Plymouth), for sufficient funds to carry out a scheme for a cottage hospital planned in Jubilee year.

The Seaside Convalescent Hospital at Seaford, Sussex, the oldest institution of its class in England, is earnestly soliciting the charitable public for a much-needed increase in the subscriptions list.

A financial re-arrangement has been effected at the Leamington Provident Dispensary, through which alteration it is hoped that 2½ per cent. be carried to the reserve fund at the end of each year.

The annual report of the Lichfield Nursing Institution and Invalid's Kitchen for 1893 has been issued, and demonstrates the great value of the organisation. It is stated that 5,572 visits have been paid during the year.

Miss Deborah Cannon has bequeathed £500 to the Stewart Institution for Lunatics, Palmeston; £300 each to the Royal Hospital for Incurables, Donnybrook, the Adelaide Hospital, Dublin, and the Monkstown Hospital.

The total cost of the new wing to the Staffordshire General Infirmary has been about £4,500, and this has been covered by subscriptions and the proceeds of the bazaar held in favour of the building funds for this much-needed extension.

A grand fancy fair, in aid of St. Joseph's Convalescent Home, was held recently at Bournemouth. Although this establishment is under the immediate direction of Roman Catholic nuns, patients are received here independent of creed.

A very successful concert, accompanied by the customary Christmas festivities, took place this year in the convalescent ward of the Alton Cottage Hospital. The patients in the hospital had a Christmas dinner supplied them by Mr. John James, of Holybourne.

A public meeting was recently convened at Pershore, to receive the report of a committee to consider the advisability of establishing a cottage hospital for that locality; Mr. Charles Ganderton having provided £500 towards the erection of the same.

The Prince of Wales has sent presents of game for the use of patients in St. Thomas's Hospital, St. George's Hospital, the Royal Eye Hospital, Southwark, the Consumption Hospital, Brompton, and the Hospital for Epilepsy and Paralysis, Regent's Park.

The Workpeople's Collection Committee of the Lancaster Infirmary does not show such a successful result in this year's report. The collectors attribute this decrease of £60 to the continued depression of trade which has lately had to be contended against in this locality.

There have been 43 patients under treatment in the Coldstream Cottage Hospital since the last annual report. District nursing has been actively carried on throughout the year by the hospital staff. The hospital continues to prove a great benefit to the neighbourhood.

The Convalescent Home in connection with the Dundee Royal Infirmary has received a substantial addition to its existing funds. Miss Harris has recently transferred to that institution stock to the value of about £3,000, which will prove a welcome addition to the revenue of the home.

The London County Council, on the recommendation of their Parliamentary Committee, have decided to petition Parliament for leave to bring in a Bill to empower the Council to purchase or acquire any areas or water rights, the possession of which might be regarded as valuable in the future.

Much discussion has taken place lately concerning the Coventry Provident Dispensary, in which the "outside" medical men desired that the privilege of membership should be regulated by the pecuniary position of the applicant. The committee of the institution, however, have not seen their way to introduce this new measure.

There is considerable antipathy in the neighbourhood of Bristol to the idea that Clift House should be used as a fever hospital for the district; and meetings consequent on this proposition are being largely attended to protest against the house alluded to being finally settled on as an infectious diseases hospital.

During the last year 811 new in-patients and 1,289 out-patients received treatment at the Cancer Free Hospital, Fulham Road. Contingently on the outlay involved on the important additions which have recently been made to this institution, an earnest appeal for fresh contributions is being urged by the authorities.

### Medical Vacancies and Appointments.

### APPOINTMENTS.

A. Asher, M.B., C.M.Edin., appointed Joint Medical Officer to the Thurso Parochial Board. S. J. Barton, M.D.Dub., appointed Medical Officer of Health for the St. Faith's Rural Sanitary District. R. Campbell, B.A., M.B.I., M.R.C.S.Eng., L.R.C.P.Lond., appointed Visiting Surgeon to the Chester General Infirmary. H. B. Densham, M.B., C.M.Edin, appointed Honorary Surgeon to the Stockton-on-Tees and Thornaby Hospital. R. B. Duncan, M.D., B.S.Dunelm, appointed Resident Medical Officer to the Newcastle-on-Tyne Workhouse. E. J. Farmer, B.A., M.B., B.Ch.Dubl., appointed Assistant Medical Officer to the Kolar Gold Fields, Mysore State, India. E. A. Farr, L.R.C.P.Lond., M.R.C.S.Eng., appointed Medical Officer of Health for the Andover Urban Sanitary District. H. Gardner, M.R.C.S.Eng., L.R.C.P.Lond., appointed Resident Medical Officer to the Chelsea Hospital for Women, Fulham Road. W. H. Gossage, M.R.C.S.Eng., L.R.C.P.Lond. (late House Surgeon), appointed House Physician to the Westminster Hospital. R. G. Griffith, M.R.C.S.Eng., appointed Chief Medical Officer to the East Indian Railway. R. Hamilton, M.B., M.R.C.S.Eng., L.R.C.P.Lond., appointed House Surgeon to the Chester General Infirmary. G. H. Hogg, M.B., C.M.Edin., appointed House Surgeon to the General Hospital, Hobart, Tasmania. C. L. Hudson, F.R.C.S., appointed Aural Surgeon at Middlesex Hospital. H. R. Kenwood M.B., C.M.Edin., L.R.C.P., D.P.H.Eng., appointed Medical Officer of Health for the Finchley Urban Sanitary District of the Barnet Union. W. W. Lake, M.R.C.S.Eng., D.P.H.Camb., appointed Medical Officer of Health for the Woking Urban Sanitary District of the Guildford Union. W. Masson, M.B., C.M.Aberd., appointed Medical Officer and Public Vaccinator to the Cottingham and Willerby District of the Sculcoates Union, Hull. R. M. Matheson, M.B., C.M.Edin., appointed House Surgeon to Nobles Hospital, Douglas, Isle of Man. C. G. May, M.D., M.R.C.P., appointed Assistant Physician to the Grosvenor Hospital for Women and Children, S.W. J. Murray, M.B., F.R.C.S., appointed Surgical Registrar to Middlesex Hospital. E. B. Ormerod, M.R.C.S., L.S.A. Lond., appointed Surgeon to employés of the Appantoo Gold Mining Company (Limited), Gold Coast Colony, South Africa. C. Parker, M.B., C.M.Edin., appointed House Surgeon to the Launceston Hospital, Tasmania. J. A. Potts, M.B.Edin., M.R.C.S., appointed Honorary Surgeon to the Ross Cottage Hospital. T. F. Ricketts, B.Sc.Lond., M.D., appointed Medical Superintendent of the Hospital Ships of the Metropolitan Asylums District. C. H. Roberts, F.R.C.S., M.B.London, M.R.C.P., appointed Casualty Physician to St. Bartholomew's Hospital. A. W. Sheperd, L.R.C.P., L.R.C.S.I., appointed Medical Officer of Health for Cowbridge. W. W. Sinclair, M.B Aberd., reappointed Senior House Surgeon to the Birmingham and Midland Eye Hospital. W. H. Webb, M.D.Durh., appointed Medical Officer of Health to the Kingsbridge Local Board. W. S. West, M.A., M.B., B.C.Cantab., appointed House Surgeon to the North-Eastern Hospital for Children, Hackney Road. P. M. Yearsley, F.R.C.S.Eng., appointed Honorary Aural Surgeon to the Farringdon General Dispensary.

### VACANCIES.

#### Resident Medical Officers.

Cheshire County Asylum, Upton, near Chester. Junior Assistant Medical Officer. Salary £120 per annum, with board, lodging, and washing. Applications and testimonials by January 15th, to Dr. Davidson, Medical Superintendent.

Liverpool Infirmary for Children, Myrtle Street, Liverpool. House Surgeon. Salary £85 per annum, with board and lodging. Applications and testimonials to be sent by January 22nd.

Liverpool Northern Hospital. Assistant House Surgeon. Salary £70 per annum, with residence and maintenance in the house. Applications and testimonials to be sent to the Chairman of Committee by January 16th.

#### Non-Resident Medical Officers.

City of Manchester. Medical Officer of Health. Salary £850 per annum. Applications and testimonials endorsed "Medical Officer of Health," to be delivered to the Lord Mayor, Town Hall, Manchester, by January 18th.

Hospital for Consumption and Diseases of the Chest. House Physicians. Applications and testimonials to the Secretary by January 19th.

Isle of Wight Union, Medical Officer for the Ryde District. Salary £110 per annum, with usual extra fees. Applications to the Clerk, Isle of Wight Union, Newport, Isle of Wight, by January 17th.

Naas Union. Clane and Timahoe Dispensary. Medical Officer. Salary £95 per annum and residence, with £15 yearly as Medical Officer of Health, registration and vaccination fees. Applications to the Assistant Honorary Secretary, Mr. J. Healy, Firmount, Clane. Election on 15th inst.

University College of South Wales and Monmouthshire. Lecturer on Materia Medica and Pharmacy. Stipend £50 a year. Applications to Ivor James, Registrar, by January 27th.

London: The Scientific Press (Limited), 428, Strand, W.C.

AS SUPPLIED TO HER MOST GRACIOUS MAJESTY.

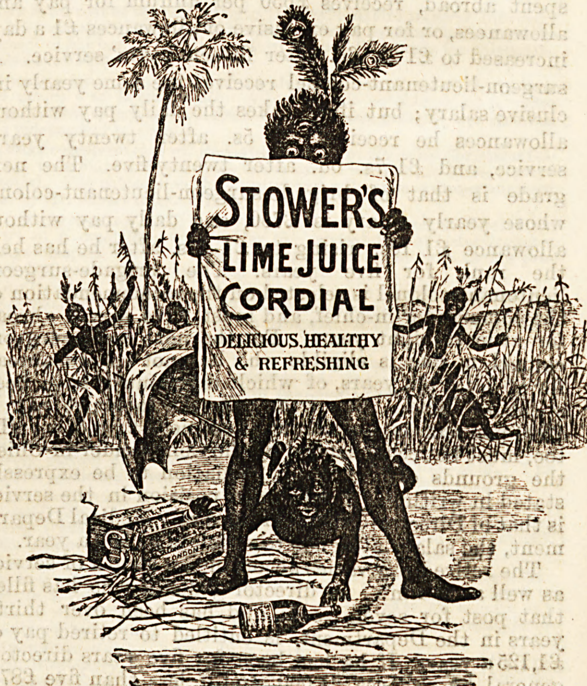

OVER 900 unsolicited testimonials from the leading Physicians and Doctors in London and the United Kingdom. Strongly recommended by the "Lancet" and "British Medical Journal." *Absolutely Pure and Free from Alcohol.*

**Samples free to Matrons and Nurses on mentioning this paper by the Proprietors,**

**A. RIDDLE & CO., 36 & 38, COMMERCIAL ST., LONDON.**

### SPECIAL TO NURSES.

### ROBINSON and CLEAVER, BELFAST,

Awarded Grand Diploma of Honour, Highest Award, Edinburgh, 1890. Two Prize Medals, Paris, 1889.

**CAMBRIC POCKET HANDKERCHIEFS.**

Children's ... ... ... ... 1/3 per dozen.

Ladies' ... ... ... ... 2/3 "

Gentlemen's ... ... ... ... 3/3 "

HEM-STITCHED.

Ladies' ... ... ... ... ... 2/9 per dozen.

Gentlemen's ... ... ... ... 3/11 "

"Cheapest handkerchiefs I have ever seen."—*Sylvia.*

"The Irish Cambrics of Messrs. ROBINSON and CLEAVER have a world-wide fame."—*The Queen.*

LINEN COLLARS AND CUFFS.

COLLARS—Ladies' 3-fold, from 3/6 per doz.; Gents' 4-fold, from 4/11 per doz.

CUFFS—For Ladies, Gentlemen, and Children from 5/11 per doz.

**NURSES' COLLARS and CUFFS**—The Sister Eva Collars, 5/6 Cuffs, 7/11 per doz.

**NURSES' APRONS**, made of fine strong linen, with hem-stitching on top, bottom and pocket, full size, 17/6 the ½-doz.

SAMPLES and ILLUSTRATED PRICE LISTS of above, and of IRISH DAMASK TABLE LINEN, POST FREE.

**ROBINSON and CLEAVER,** By Special Appointments to the Queen and Empress Frederick of Germany. **BELFAST.**

PLEASE NAME THIS PUBLICATION.

Demy 8vo., 200 pp., cloth extra, price 3s. 6d.,

**SURGICAL WARD-WORK AND NURSING**: A practical Manual of Clinical Instruction for Nurses and Students. This book will be found especially useful to beginners, to whom its simple description of elementary principles and treatment in surgery will render it invaluable. By ALEXANDER MILES, M.D. (Edin.), C.M., F.R.C.S.E. Over one hundred illustrations.

LIST OF CHAPTERS.

SECTION I.—ANTISEPTIC SURGERY. Chap. 1. General Principles of Antiseptic Surgery—2. Antiseptic Lotions—3. Antiseptic Powders and Unguents—4. Materials for Dressings—5. Lotion Basins—6. Ward Table and Dressing Tray—7. Antiseptic Dressing—8. A Spray Dressing—9. Management of Surgical operations—10. The Lotion Table—11. Dressings Table—Anæsthetics—12. Preparation of Patient for Operation—13. Treatment after Operation—14. Nursing of Special Cases after Operation.

SECTION II.—THE USE OF REST IN SURGERY. 15. Extension Apparatus—16. Plaster and Starch Cases—17. Appliances for Joint Affections—18. Appliances for Fractured Bones.

SECTION III.—BANDAGING. 19. Bandaging—20. Special Bandages—21. Special Bandages (*continued*)—22. Sspecial Bandages (*continued*).

SECTION IV.—SURGICAL INSTRUMENTS AND APPLIANCES. 23. General Instruments—24. Instruments for Hæmorrhage—25. Knives—26. Saws—27. Bone Instruments—28. Instruments used in Trephining—29. Mouth Instruments, etc.—30. Intestinal Instruments—31. Urethral Instruments—32. Lithotomy and Lithotrity Instruments—33. Laryngeal Instruments—34. Aural, Nasal, and Ophthalmic Instruments—35. Gynæcological Instruments.

"Will furnish much-needed guidance to the student and the nurse in the early stages of their apprenticeship."—*Times.*

"This is a fully illustrated handbook for the use of junior students of medicine and nurses, which should prove of very great value, Dr. Miles being unquestionably an authority on the subject of which he writes."—*Publishers' Circular.*

Just ready, Important New Text-Book of Ophthalmic Nursing.
Crown 8vo., cloth gilt, pp. 200, profusely illustrated, and with many woodcuts, price 3s. 6d., post free,

**OPHTHALMIC NURSING.** By SYDNEY STEPHENSON, M.B., F.R.C.S.E., Surgeon to the Ophthalmic School, Hanwell, W., &c., &c. A complete text-book on this branch of nursing, illustrated by numbers of original figures, and containing a glossary of terms and list and description of instruments used in ophthalmic nursing.

As yet no book has been published dealing with nursing in diseases of the eye. As a text-book, "Ophthalmic Nursing" will be invaluable to nurses desiring to qualify in this particular branch of nursing. The managers of Ophthalmic Institutions are invited to adopt this work as a text-book for their nursing staffs. The book contains a frontispiece, "Vertical Section of the Eye-ball and Eye-lids," and sixty-one other illustrations It is concisely yet exhaustively written, and its method of treatment and style will render it especially serviceable to the Nursing Profession.

THE LANCET says: "The work now before us gives the result of his experience to those who have charge of Ophthalmic patients, and will prove useful. A good account is given of the proper arrangements and preparations that should be made for operations, with the various modes of bandaging one or both eyes. The work may very advantageously be studied by nurses and clinical clerks, or dressers who are about to enter the Ophthalmic service of a hospital."

NURSING NOTES says: "A book on Ophthalmic Nursing has been a real need which is now well supplied. . . . . Those contemplating going in for Ophthalmic Nursing should study this useful work. . . . . One of the most useful books for nurses we have seen for a long time."

THE OPHTHALMIC REVIEW says: "The author has evidently endeavoured to write in language which will be intelligible to nurses of all grades, and we congratulate him on the clearness with which his statements are expressed. . . . . Chapters II. and III., upon 'The Germ Theory of Disease' and 'Contagion and Infection,' are good, and the latter contains useful rules for the avoidance by nurses and others of the contagion of eye disease."

"Will prove of inestimable value."—*Publishers' Circular.*

Third Edition, enlarged and partly re-written, cloth gilt, crown 8vo., about 200 pp., price 2s. 6d.,

**HOSPITAL SISTERS AND THEIR DUTIES.** By EVA C. E. LÜCKES, Matron to the London Hospital. Every matron, sister, or nurse who aspires to become a sister should read the valuable advice and information contained in this book. The author's experience as matron of London's largest hospital peculiarly qualifies her to speak on this subject.

"We cordially recommend the work to all hospital authorities as well as to the class for whose special benefit it is more immediately intended."—*Daily Chronicle.* "On every page we find evidence of Miss Lückes' thorough acquaintance with all the details of the nursing department of hospital management."—*Lancet.* "It is well worthy of study for those to whom it is addressed, and for all who are interested in the matter of nursing."—*New York Medical Journal.*

Second Edition, enlarged, demy 8vo., profusely illustrated, nearly 200 pp., cloth. Important Text-Book for Asylum Attendants, price 2s. 6d., post free,

**MENTAL NURSING.** A Text-Book specially designed for the instruction of Attendants on the Insane. By WILLIAM HARDING, M.B.

"Nothing could be better devised to serve as a text-book. The lectures are so simple and so instructive that they cannot fail to impress and interest readers. . . . Of great value to all nurses. . . . No institution should fail to supply their officials with a copy of the work."—*Local Government Journal.* "The book is written in a clear, easily understood style."—*Glasgow Medical Journal.*

Demy 8vo., 200 pp., Fourth Thousand, profusely illustrated with Copyright Portraits and Drawings, price 2s. 6d., post free,

**HOW TO BECOME A NURSE: And How to Succeed.** Compiled by HONNOR MORTEN, Author of "Sketches of Hospital Life," "The Nurses' Dictionary," &c. A complete collection of information of every kind such as persons desiring to enter nursing can want to know. General advice, useful hints, and full list of hospitals, nursing schools, and other institutions to which intending nurses can apply for admission as probationers. Also terms, fees, and every other detail.

"This book . . . must prove a perfect godsend."—*British Medical Journal.*

London: THE SCIENTIFIC PRESS (Limited), 428, Strand, W.C.

In Two Series, specially designed and ruled.

**ACCOUNT BOOKS FOR INSTITUTIONS.** Designed by HENRY C. BURDETT. Ruled in accordance with the uniform system of accounts advocated by Metropolitan Hospital Sunday Fund, and prepared for the convenience and assistance of Secretaries who present annual returns for participation in the Hospital Sunday Fund Grants.

These Books are ruled so that the various descriptions of Receipts and Expenditure, &c., &c., may be entered uniformly under the special headings and in the columns prepared for them.

There are TWO SERIES, No. 1 being for the larger Institutions, and No. 2 for Cottage Hospitals and Small Institutions. The sets are sold complete, at a specially reduced price; or each book may be had separately, at the prices set out below :—

**SERIES No. 1.—FOR HOSPITALS AND INSTITUTIONS.**

(Any of these Books may be purchased separately; a reduction is made if the complete set is taken.)

1. Analysis Journal. Royal, 350 pp. Divided into seven sections, with parchment tags. Headings as follows :—
   A. Maintenance.
   1. Provisions. 5. Rent.
   2. Surgery & Dispensary. 6. Salaries.
   3. Domestic. 7. Miscellaneous Expenses
   4. Establishment Charges.
   B. Administratation.
   1. Management. 2. Finance.
   (Uniform with Headings in Income and Expenditure Table.) Each Heading is ruled with pages containing 15 columns. Price £3.
2. Cash Book. Foolscap, 300 pp., printed Headings, specially ruled. Price £1.
3. Cash Analysis and Receipt Book. Foolscap, extra width, 300 pp., ruled, with 15 columns, printed Headings identical with Income and Expenditure Table. Price £1 5s.
4. Secretary's Petty Cash Book. 300 pp., foolscap, ruled with 13 columns, printed Headings identical with Income and Expenditure Table. Price £1.
5. List or Register of Annual Subscribers. Double foolscap, 300 pp., ruled with 10 columns, printed Headings for the years 1893-1900 inclusive. Price £1 15s.
6. Alphabetical Register Book. Foolscap, 300 pp., ruled, with date, name and address, and cash colums, cut into alphabetical sections, with totals at end for annual balances. Price £1 6s.
7. Subscription Register. Foolscap, ruled with 8 columns, printed Headings for date when subscriptions become due, name and address, and cash columns for years 1893 to 1898 inclusive. Price £1 1s.
8. Linen Register. Foolscap long quarto, 300 pp., ruled with columns for Stock, Condemned, Remaining, and Issued Linen, also total in Ward and Remarks, also printed lists of Hospital Linen. Price 8s. 6d.
9. Income and Expenditure Table. Basis of uniform system. For yearly balances and statements. Royal. Price 6d. per sheet, or 5s. per dozen.
10. Special Appeal Account Table. Foolscap. Price 3d. per sheet, or 2s. 6d. per dozen.

Price of Set Complete, £10 Net.

**SERIES No. 2.—FOR COTTAGE HOSPITALS AND SMALLER INSTITUTIONS.**

1. Cash Analysis, Receipt, and Expenditure Book. Double foolscap, 300 pp., ruled with 18 columns, printed Headings identical with Income and Expenditure Table, specially prepared for Cottage Hospitals and Small Institutions. Price £1 15s.
2. Secretary's Petty Cash Book.—300 pp., foolscap, ruled with 13 columns, printed headings identical with Income and Expenditure Table. Price £1.
3. Income and Expenditure Table. Basis of uniform system. For yearly balances and statements. Royal. Price 6d. per sheet, or 5s. per dozen.
4. Linen Register. Foolscap, 300 pp., ruled with columns for Stock, Condemned, Remaining, and Issued Linen, also total in Ward and Remarks, also printed lists of Hospital Linen. Price 8s. 6d.
5. List or Register of Annual Subscribers. Foolscap. 300 pp., ruled with columns for the years 1893-1900 inclusive. Price £1.
6. Special Appeal Account Tables. Foolscap. Price 3d. per sheet, or 2s. 6d. per dozen.

Price of Set Complete, £4 Net.

The books are all uniformly bound in half basil, with gilt letterings, and are ruled on best paper, and in every way prepared for practical use at the offices of Institutions.

Demy 8vo., profusely illustrated with Specimen Tables, Index of Classification, Forms of Tender, &c., cloth extra, price 6s., post free.

**ACCOUNTS, AUDIT, AND TENDERS; the Uniform System of,** For Hospitals and Institutions, with Certain Checks upon Expenditure. Also the Index of Classification. Compiled by a Committee of Hospital Secretaries, and adopted by a General Meeting of the same, 18th January, 1892. And certain Tender and other Forms for securing Economy. By HENRY C. BURDETT.

This is a hand-book to the account books, explaining their nature and uses, the method of keeping them, and the general principles upon which they were designed.

All having to do with hospital accounts will find much valuable information in this hand-book.

Demy 8vo., about 400 pp., illustrated. In the Press.

**MEDICAL HISTORY FROM THE EARLIEST TIMES.** By E. T. WITHINGTON, M.A., M.B., Oxon.

An important and comprehensive history of medicine; likely to be of great use and interest to the profession, and to students.

The work, which is in active preparation, will be embellished with some striking copies of ancient inscriptions.

Demy 8vo., cloth gilt, pp. 260. Price 3s. 6d.

**ART OF FEEDING THE INVALID.** By a Medical Practitioner and a Lady Professor of Cookery.

This important work fully and clearly describes the nature and tendency of diseases most frequently met with, and gives a general outline upon which the diet of the invalid should be regulated. Corresponding with the chapters are lists of new and original recipes, which are intended for daily use and reference.

The labour and anxiety of providing suitable diet for invalids will, by the use of this book, be reduced to a minimum.

Among the subjects treated are the following :—The Feeding of Invalids generally; Food; Food in Acute Febrile Diseases, in Convalescence and Chronic Illness, in Indigestion, in Gout, in Constipation and Diarrhœa, in Liver and Kidney Diseases, in Diabetes, in Tuberculosis and Consumption, in Corpulence, and for Children.

"The Art of Feeding the Invalid" has the approval of Members of the Faculty, its Medical author being widely known as the author of a standard text-book.

The Recipes are entirely new, having been especially written by a Professional Cook who holds diplomas and has had vast experience as a lecturer.

It has been found a useful work to place in the hands of nurses and patients as a guide during the doctor's absence.

